# Illusion of filtration: Evidence from tobacco industry documents

**DOI:** 10.18332/tid/166093

**Published:** 2023-06-23

**Authors:** K. Michael Cummings, Avery Roberson, Dana M. Carroll, Irina Stepanov, Dorothy Hatsukami, Vaughan W. Rees, Richard J. O’Connor

**Affiliations:** 1 Department of Psychiatry and Behavioral Sciences, Medical University of South Carolina, Charleston, United States; 2 Division of Environmental Health Sciences, School of Public Health, University of Minnesota, Minneapolis, United States; 3 Department of Psychiatry and Behavioral Sciences, University of Minnesota, Minneapolis, United States; 4 Department of Social and Behavioral Sciences, Harvard T.H. Chan School of Public Health, Boston, United States; 5 Department of Health Behavior, Roswell Park Comprehensive Cancer Center, Buffalo, United States

**Keywords:** cigarette smoking, cancer risk, consumer perception, cigarette design

## Abstract

**INTRODUCTION:**

We compared the design features of popular filtered and non-filtered cigarettes sold in the United States between 1960 and 1990, to assess the relationship between cigarette filter and tobacco weight.

**METHODS:**

We analyzed data on the design features of six popular filtered and three non-filtered cigarette brands sold in the US including the weight of tobacco used provided in the Cigarette Information Reports produced by Philip Morris Tobacco Company between 1960 and 1990. We also collected information on other design features such as stick length and circumference, the percentage of reconstituted tobacco in the blend, among other product parameters. We used joinpoint regression to test for trends in outcome variables for each brand assessed between 1960 and 1990.

**RESULTS:**

In all years, filtered cigarettes had less tobacco by weight compared to non-filtered cigarettes. The lower average weight of tobacco found in filtered cigarettes appears to be due to a combination of factors including stick and filter length, and the amount of reconstituted tobacco in the blend. The average percentages of total alkaloids and expanded tobacco increased over time but were similar between filtered and non-filtered brands.

**CONCLUSIONS:**

While various design features of popular filtered and non-filtered brands changed between 1960 and 1990, the observed reduction in tobacco weight among filtered brands was perhaps the most salient in terms of disease risk. Less tobacco in a filtered cigarette calls into question the presumed exclusive role of cigarette filter tips in the reduced health risks of filtered versus non-filtered cigarette smoking.

## INTRODUCTION

The cigarette filter rose to prominence in the 1950s and 1960s as a response to growing concerns about the health risks of smoking^1-3^. In 1952, cigarettes with a filter tip accounted for only 1.4% of sales, but increased to over 40% of the market by the end of the 1950s^4^. Between 1965 and 1990, the share of non-filtered cigarettes declined from 36% in 1965 to 5% in 1990^5^. In 2021, non-filtered cigarettes accounted for 0.2% of cigarettes sold in the US^5^.

Early filtered cigarette brands made explicit health claims, leading many consumers to believe there was a health benefit to adding a filter to a cigarette; this perception is still prevalent today^1-4,6-13^. Additionally, epidemiologic studies conducted since the 1960s have also reported lower risk of lung cancer and all-cause mortality attributable to the use of a filter tip on cigarettes, reinforcing a widely held view that the filter tip on a cigarette reduces the risks associated with smoking^14-23^.

An important limitation of past epidemiologic studies is that they failed to adjust for design features of cigarettes that could potentially explain the purported reduction in smoking-related health risks that had been attributed to the cigarette filter. One such design feature is the amount of tobacco available to be burned in the cigarette stick itself. The presumed advantage of the cigarette filter is the reduced availability of toxic smoke constituents to which a consumer might otherwise be exposed^14-16^. To better account for differences in toxicant exposure on health risks, some epidemiologic studies attempted to adjust for the tar and nicotine deliveries of filtered and non-filtered cigarettes by using standardized machine measured tar and nicotine yields reported to the FTC^21^.

However, what was not fully appreciated by researchers or the public, was that the filter tip itself influences smoking behaviors^24^. For example, the role of compensation (e.g. puffing differently and/or smoking more cigarettes) and the apparent absence of any real health benefit from the addition of a filter tip on a cigarette was noted by Philip Morris as early as 1961 when the Director of Research & Development observed that ‘all too often the smoker who switches to a hi-fi (high filtration) cigarette winds up smoking more units in order to provide himself with the same delivery which he had before’^25^. A similar observation – this time regarding the actual presence or absence of a filter – was noted in a 1976 memo from a Brown & Williamson internal business record, which stated: ‘the smoker of a filtered cigarette was getting as much or more nicotine and tar as he would have gotten from a regular (unfiltered) cigarette. He had abandoned the regular cigarette, however, on the ground of reduced risk to health’^26^.

Other cigarette design features have not been considered to date in non-industry research of the risks of filtered and non-filtered cigarettes because the product features themselves were considered propriety information by manufacturers and were shared neither with members of the research community nor the public. However, access to previously secret cigarette company documents available on the Truth Tobacco document website (https://www.industrydocuments.ucsf.edu/tobacco) has opened the door to examining the different product design features of cigarette brands.

In this study, we accessed Research & Development reports produced by Philip Morris Tobacco Company between 1960 and 1990 that carefully monitored the design features of cigarette brands sold in the United States^27-33^. The main focus of our descriptive analysis is on the weight of tobacco in the cigarette stick because the amount of tobacco burned is likely to be a significant factor in determining on a per cigarette basis the level of exposure to cancer causing chemicals in tobacco smoke^14,23-26^. However, we also assessed other design features to explore how different cigarette brands were modified over time and to examine differences in the characteristics of popular filtered and non-filtered cigarettes.

## METHODS

We utilized a sample of Cigarette Information Reports (CIRs) produced by Philip Morris to compare the weight of tobacco used in popular filtered and non-filtered cigarette brands over a 30-year period between 1960 and 1990. These reports appear to have been produced monthly, allowing Philip Morris management to closely monitor the design features and performance characteristics of their own brands as well as their competitors.

While there are hundreds of brands and brand styles reported in the CIRs, we reviewed^27-33^, we chose to narrow our focus to the bestselling filtered and non-filtered brands as reported in the 1960s. We selected one CIR from each of the following years: 1960, 1965, 1970, 1975, 1980, 1985, and 1990, and compared the weight of tobacco found in nine popular filtered and non-filtered cigarette brands. We selected six filtered brands, three of which were regular/non-mentholated flavored (i.e. Marlboro, Winston, Kent) and three were menthol flavored (i.e. Salem, Kool, Newport). We also selected three non-filtered/non-mentholated brands that were part of the CIRs reports over the duration of the examined time period, for comparison (i.e. Camel, Lucky Strike, and Philip Morris). All of the filtered cigarettes were 85 mm in length while the non-filtered cigarette brands were 70 mm in length.

We describe the following cigarette design features for each cigarette brand by year: cigarette stick length (mm) and circumference (mm), weight of tobacco (g), percentage of reconstituted tobacco in the blend (reported from 1965 to 1990), percentage of expanded tobacco in the blend (reported from 1975 to 1990), the percentage of alkaloids in the tobacco, and the total particulate matter (TPM mg/cigarette), and nicotine yields (mg/cigarette). The TPM and nicotine deliveries in the 1960s are based on testing performed by Philip Morris while TPM and nicotine yields reported from 1970 to 1990 are based on FTC reports. For the filtered cigarette brands, we also report on filter length and percentage of filter dilution (i.e. a measure indicative of the use of vent holes in the filter tip allowing air to dilute the smoke as it travels through the filter).

In addition to descriptive statistics done in SAS 9.4^34^, we used joinpoint regression to test for trends in outcomes variables for each brand assessed between 1960 and 1990. Joinpoint computes the average annual percentage change (AAPC) and 95% confidence intervals (CIs) for each outcome, testing to determine if the average trend is significantly different than zero at the alpha = 0.05 level^35^. We also used repeated measures analysis of variance (ANOVA) to compare filtered versus non-filtered brands on selected outcomes such as weight of tobacco.

## RESULTS

Tables 1–3 show the design features of nine popular filtered and non-filtered cigarette brands sold in the US between 1960 and 1990, and the results of jointpoint regressions for each outcome. Joinpoint regression analyses show significant changes in key design parameters over time. For example, the weight of tobacco was reduced significantly in all brands between 1960 and 1990, while the percentage of reconstituted tobacco, expanded tobacco and alkaloids in the tobacco increased. In the six filtered brands, the length of the filter and the percentage of filter dilution increased significantly, while the length of the tobacco stick and average TPM levels decreased between 1960 and 1990. The average TPM levels of the three non-filtered brands did not change significantly between 1960 and 1990.

**Table 1:** Selected features of three filtered, 85 mm, non-menthol cigarette brands, 1960–1990

*Cigarette brand*	*Product features*	*Year of assessment*							*1960–1990 AAPC % (95% CI)*
		*1960*	*1965*	*1970^†^*	*1975*	*1980*	*1985*	*1990*	
**Marlboro**	Weight of tobacco (g)	0.88	0.85	0.84	0.78	0.76	0.75	0.75	-0.6 (-0.7 – -0.5)*
	Stick length (mm)	65.0	64.4	64.4	63.6	63.3	62.7	63.2	-0.1 (-0.2 – -0.0)*
	Stick circumference (mm)	25.5	25.3	25.3	25.0	24.9	25.0	25.0	-0.1 (-0.1 – -0.0)*
	Reconstituted tobacco (%)	NP	16.0	17.0	18.0	22.0	21.0	22.0	1.4 (0.8 – 1.9)*
	Expanded tobacco (%)	NP	NP	NP	3.0	11.0	12.0	12.0	8.9 (-5.5 – 25.6)
	Total alkaloids (%)	1.7	1.7	1.7	1.6	1.6	1.9	2.1	0.7 (0.4 – 1.0)*
	Filter length (mm)	20.0	20.1	20.0	20.9	21.0	21.2	21.0	0.2 (0.1 – 0.3)*
	Filter dilution (%)	0	0	0	0	9.0	9.0	11.0	2.0 (0.2 – 3.9)*
	TPM (mg/cigarette)	27.0	22.0	20.0	17.0	16.0	16.0	16.0	-1.7 (-1.9 – -1.5)*
	Nicotine (mg/cigarette)	1.4	1.2	1.3	1.0	1.1	1.1	1.1	-0.8 (-1.6 – 0.1)
	Sales (billion cigarettes)	22.0	27.1	45.9	72.5	70.9	66.6	27.1	1.2 (-1.6 – 4.4)
**Winston**	Weight of tobacco (g)	0.94	0.92	0.82	0.79	0.77	0.77	0.71	-0.9 (-1.4 – -0.4)*
	Stick length (mm)	68.0	67.7	64.8	63.6	63.3	63.0	63.0	-0.3 (-0.3 – -0.2)*
	Stick circumference (mm)	25.4	25.3	25.0	25.0	24.9	24.9	25.0	-0.1 (-0.1 – -0.0)*
	Reconstituted tobacco (%)	NP	17.0	23.0	22.0	25.0	23.0	25.0	1.2 (-0.6 – 3.0)
	Expanded tobacco (%)	NP	NP	NP	12.0	14.0	16.0	16.0	2.0 (0.3 – 3.8)*
	Total alkaloids (%)	1.8	1.6	1.7	1.7	1.7	1.8	2.1	0.6 (0.2 – 0.9)*
	Filter length (mm)	17.0	17.0	20.0	21.0	20.9	21.0	21.0	0.8 (0.5 – 1.1)*
	Filter dilution (%)	0	0	0	0	0	14.0	8.0	-
	TPM (mg/cigarette)	24.0	22.0	19.0	19.0	15.0	16.0	16.0	-1.4 (-1.8 – -1.0)*
	Nicotine (mg/cigarette)	1.5	1.3	1.3	1.3	1.0	1.1	1.1	-1.1 (-1.9 – -0.2)*
	Sales (billion cigarettes)	52.4	72.0	66.6	67.6	45.4	32.5	18.7	-3.6 (-4.4 – -2.7)*
**Kent**	Weight of tobacco (g)	0.83	0.81	0.83	0.76	0.73	0.73	0.75	-0.5 (-0.8 – -0.1)*
	Stick length (mm)	68.0	64.8	65.0	64.4	63.3	63.0	63.5	-0.2 (-0.3 – -0.1)*
	Stick circumference (mm)	25.1	25.1	25.1	25.0	25.1	25.2	24.9	-0.0 (-0.0 – 0.0)
	Reconstituted tobacco (%)	NP	7.0	11.0	21.0	21.0	19.0	22.0	4.3 (0.0 – 8.6)*
	Expanded tobacco (%)	NP	NP	NP	11.0	12.0	12.0	11.0	0.0 (-2.3 – 2.4)
	Total alkaloids (%)	1.3	1.4	1.4	1.4	1.8	1.9	2.0	1.6 (1.0 – 2.1)*
	Filter length (mm)	17.0	19.9	19.7	20.2	21.0	21.0	20.8	0.6 (0.2 – 1.0)*
	Filter dilution (%)	0	0	0	0	0	23.0	18.0	-
	TPM (mg/cigarette)	23.0	18.0	15.0	15.0	13.0	12.0	12.0	-2.2 (-2.6 – -1.7)*
	Nicotine (mg/cigarette)	1.0	0.9	1.0	1.0	1.0	0.9	0.9	-0.2 (-0.9 – 0.4)
	Sales (billion cigarettes)	38.2	30.5	21.5	17.9	7.9	4.6	2.4	-8.9 (-9.5 – -8.4)*

† From 1970–1990, tar and nicotine values are based on FTC measured levels. AAPC: average annual percent change.

* Indicates that the AAPC is significantly different from zero at the alpha = 0.05 level. TPM: total particulate matter. NP: not reported

**Table 2:** Selected features of three filtered, 85 mm, menthol cigarette brands, 1960–1990

*Cigarette brand*	*Product features*	*Year of assessment*							*1960–1990 AAPC % (95% CI)*
		*1960*	*1965*	*1970^†^*	*1975*	*1980*	*1985*	*1990*	
**Salem**	Weight of tobacco (g)	0.88	0.93	0.83	0.79	0.79	0.75	0.71	-0.8 (-1.1 – -0.5)*
	Stick length (mm)	68.0	67.9	64.8	63.3	63.2	62.7	63.1	-0.3 (-0.4 – -0.2)*
	Stick circumference (mm)	25.4	25.3	25.0	25.0	24.9	25.0	25.1	-0.0 (-0.1 – -0.0)*
	Reconstituted tobacco (%)	NP	19.0	21.0	20.0	23.0	24.0	25.0	1.1 (0.7 – 1.5)*
	Expanded tobacco (%)	NP	NP	NP	12.0	13.0	16.0	16.0	2.2 (1.0 – 3.3)*
	Total alkaloids (%)	1.7	1.8	1.7	1.8	1.8	1.9	2.3	0.9 (0.5 – 1.2)*
	Filter length (mm)	17.0	17.0	20.0	20.9	20.8	21.0	21.0	0.8 (0.5 – 1.1)*
	Filter dilution (%)	0	0	0	0	17.0	0	0	-
	TPM (mg/cigarette)	28.0	22.0	19.0	18.0	14.0	15.0	16.0	-1.7 (-2.4 – -1.3)*
	Nicotine (mg/cigarette)	1.5	1.3	1.4	1.3	1.1	1.0	1.0	-1.4 (-2.3 – -0.5)*
	Sales (billion cigarettes)	35.1	45.4	35.1	34.4	17.7	12.3	7.4	-5.5 (-6.4 – -4.7)*
**Kool**	Weight of tobacco (g)	0.86	0.81	0.77	0.76	0.75	0.76	0.71	-0.6 (-0.7 – -0.4)*
	Stick length (mm)	68.0	65.2	63.0	62.9	62.9	62.9	62.9	-0.3 (-0.3 – -0.2)*
	Stick circumference (mm)	24.9	25.2	25.1	25.1	24.9	25.1	25.0	-0.0 (-0.1 – 0.1)
	Reconstituted tobacco (%)	NP	8.0	8.0	13.0	11.0	12.0	13.0	2.0 (0.7 – 3.4)*
	Expanded tobacco (%)	NP	NP	NP	0	0	11.0	11.0	0.0 (0.0 – 0.0)
	Total alkaloids (%)	1.9	2.1	2.0	1.9	2.1	2.2	2.5	0.7 (0.2 – 1.1)*
	Filter length (mm)	17.0	19.8	20.8	20.9	21.0	21.0	20.9	0.6 (0.5 – 0.8)*
	Filter dilution (%)	0	0	0	0	0	0	0	-
	TPM (mg/cigarette)	30.0	23.0	17.0	16.0	15.0	16.0	16.0	-2.2 (-2.5 – -1.9)*
	Nicotine (mg/cigarette)	1.6	1.4	1.4	1.2	1.1	1.1	1.1	-1.2 (-1.5 – -1.0)*
	Sales (billion cigarettes)	10.9	20.3	34.8	46.7	33.7	23.7	12.4	0.5 (-1.1 – 2.1)
**Newport**	Weight of tobacco (g)	0.83	0.83	0.82	0.75	0.73	0.72	0.72	-0.6 (-1.0 – -0.2)*
	Stick length (mm)	68.0	67.7	64.9	64.5	63.4	63.1	63.2	-0.3 (-0.3 – -0.2)*
	Stick circumference (mm)	25.0	25.1	25.0	25.0	25.1	25.1	25.0	0.0 (-0.0 – 0.0)
	Reconstituted tobacco (%)	NP	7.0	11.0	21.0	20.0	20.0	22.0	4.4 (-0.0 – 8.9)
	Expanded tobacco (%)	NP	NP	NP	11.0	11.0	12.0	11.0	0.2 (-0.8 – 1.1)
	Total alkaloids (%)	1.5	1.6	1.5	1.7	2.0	2.1	2.3	1.4 (1.2 – 1.7)*
	Filter length (mm)	17.0	17.0	19.8	19.9	20.9	21.1	21.0	0.7 (0.5 – 1.0)*
	Filter dilution (%)	0	0	0	0	0	0	0	-
	TPM (mg/cigarette)	24.0	19.0	19.0	17.0	17.0	17.0	17.0	-1.0 (-1.4 – -0.6)*
	Nicotine (mg/cigarette)	1.0	1.0	1.2	1.2	1.3	1.2	1.3	0.9 (-0.0 – 1.8)
	Sales (billion cigarettes)	5.2	8.7	3.7	5.1	6.3	9.1	9.1	1.7 (-1.0 – 4.4)

† From 1970–1990, tar and nicotine values are based on FTC measured levels. AAPC: average annual percent change.

* Indicates that the AAPC is significantly different from zero at the alpha = 0.05 level. TPM: total particulate matter. NP: not reported

**Table 3:** Selected features of three non-filtered,70 mm, non-menthol cigarette brands, 1960–1990

*Cigarette brand*	*Product features*	*Year of assessment*							*1960–1990 AAPC % (95% CI)*
		*1960*	*1965*	*1970^†^*	*1975*	*1980*	*1985*	*1990*	
**Camel**	Weight of tobacco (g)	0.95	0.96	0.89	0.87	0.84	0.81	0.77	-0.7 (-0.9 – -0.5)*
	Stick length (mm)	70.0	70.1	70.1	69.6	69.1	68.9	69.1	-0.1 (-0.1 – 0.0)
	Stick circumference (mm)	25.3	25.5	25.1	25.1	25.1	25.0	25.0	-0.1 (-0.1 – -0.0)*
	Reconstituted tobacco (%)	NP	10.0	11.0	12.0	16.0	14.0	15.0	-0.1 (-0.1 – -0.0)*
	Expanded tobacco (%)	NP	NP	NP	9.0	13.0	17.0	16.0	4.1 (-1.0 – 9.5)
	Total alkaloids (%)	1.7	1.7	1.7	1.9	2.1	2.1	2.2	1.0 (0.7 – 1.3)*
	TPM (mg/cigarette)	28.0	24.0	24.0	25.0	19.0	19.0	22.0	-1.0 (-2.5 – 0.5)
	Nicotine (mg/cigarette)	1.5	1.3	1.5	1.6	1.4	1.3	1.4	-0.2 (-0.8 – 0.4)
	Sales (billion cigarettes)	66.3	50.1	28.9	21.1	14.1	10.4	6.3	-7.5 (-8.0 – -7.0)*
**Lucky Strike**	Weight of tobacco (g)	1.00	0.95	0.92	0.89	0.89	0.85	0.89	-0.4 (-0.6 – -0.3)*
	Stick length (mm)	70.0	70.2	69.9	69.8	69.8	68.8	69.2	-0.1 (-0.1 – -0.0)*
	Stick circumference (mm)	25.6	25.5	24.9	25.0	24.9	25.0	25.0	-0.1 (-0.1 – -0.0)*
	Reconstituted tobacco (%)	NP	7.0	13.0	15.0	16.0	17.0	20.0	-0.1 (-0.1 – -0.0)*
	Expanded tobacco (%)	NP	NP	NP	3.0	6.0	11.0	11.0	9.4 (1.3 – 18.3)*
	Total alkaloids (%)	1.6	1.6	1.6	1.6	1.6	1.9	2.1	1.0 (0.8 – 1.1)*
	TPM (mg/cigarette)	31.0	20.0	27.0	28.0	24.0	23.0	24.0	-0.4 (-1.7 – 0.8)
	Nicotine (mg/cigarette)	1.5	1.1	1.7	1.6	1.5	1.5	1.5	0.4 (-1.0 – 1.7)
	Sales (billion cigarettes)	42.1	27.0	15.2	7.9	5.8	3.7	2.1	-9.5 (-10.2 – -8.8)*
**Philip Morris**	Weight of tobacco (g)	0.98	0.95	0.92	0.84	0.84	0.81	NP	-0.8 (-1.0 – -0.6)*
	Stick length (mm)	70.0	70.3	69.6	69.8	69.9	70.0	NP	-0.0 (-0.1 – 0.0)
	Stick circumference (mm)	25.4	25.1	25.3	24.8	24.7	24.8	NP	-0.1 (-0.2 – -0.0)*
	Reconstituted tobacco (%)	NP	12.0	16.0	15.0	19.0	17.0	NP	-0.1 (-0.2 – -0.0)*
	Expanded tobacco (%)	NP	NP	NP	4.0	13.0	15.0	NP	14.1 (5.9 – 23.1)*
	Total alkaloids (%)	1.8	1.6	1.8	1.6	1.7	1.9	NP	0.2 (-1.3 – 1.7)
	TPM (mg/cigarette)	30.0	22.0	23.0	21.0	20.0	22.0	NP	-1.1 (-2.6 – 0.4)
	Nicotine (mg/cigarette)	1.5	1.4	1.5	1.1	1.4	1.4	NP	-0.4 (-1.7 – 1.0)
	Sales (billion cigarettes)	5.5	2.0	0.7	0.3	0.2	0.1	NP	-14.7 (-17.8 – -11.6)*

† From 1970–1990, tar and nicotine values are based on FTC measured levels. AAPC: average annual percent change.

* Indicates that the AAPC is significantly different from zero at the alpha = 0.05 level. TPM: total particulate matter. NP: not reported

**Figure 1:**
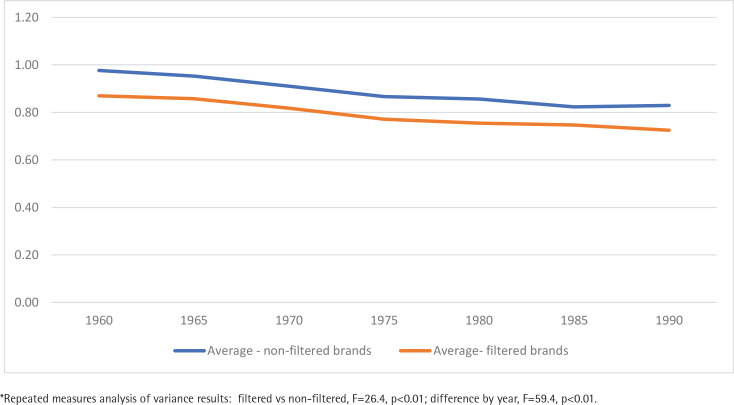
Average weight of tobacco (g) for six filtered and three non-filtered cigarette brands, 1960–1990*

Figure 1 displays the trend in the average weight of tobacco in filtered and non-filtered brands between 1960 and 1990. The weight of tobacco in filtered and non-filtered cigarettes declined in parallel between 1960 and 1990 but was significantly lower in filtered compared to non-filtered brands (p<0.01). Supplementary file Figures 1–6 display differences over time between filtered and non-filtered brands for other design parameters. Analysis of variance revealed a statistically significant main effect of filter (vs non-filtered) on measures of percentage of reconstituted tobacco, length of the tobacco stick, TPM, and nicotine level. No main effect of filter was observed on percentage of alkaloids and expanded tobacco, but these measures did increase significantly in all cigarette brands over time. Supplementary file Figure 7 shows that in filtered cigarette brands the average length of the tobacco stick decreased while the length of the filter increased between 1960 and 1990.

## DISCUSSION

The findings from this study reveal that many of the design features of popular filtered and non-filtered cigarette brands changed between 1960 and 1990. These findings have implications for studying the health risks of cigarette smoking since modifications in the design parameters of a given cigarette brand may, and likely do, influence the type and level of exposures to toxic chemicals in the tobacco smoke.

For the purposes of this descriptive analysis, we focused on changes in the weight of tobacco found in filtered and non-filtered cigarettes because this design parameter would be expected to influence exposure to the carcinogens found in the smoke (i.e. at similar smoking intensity, the more tobacco burned the more carcinogens produced). We documented that there was consistently less tobacco available to be burned in popular filtered compared to non-filtered cigarette brands. This finding is consistent with lower machine-measured TPM deliveries of filtered cigarettes compared to non-filtered. The lower average weight of tobacco found in filtered cigarettes appears to be due to a combination of factors including reductions in the length of the tobacco stick and corresponding increase in filter length, filter dilution possibly, and the greater use of reconstituted tobacco in the blend of filtered compared to non-filtered cigarettes.

### Limitations

As in any study, there are limitations that readers should be cautioned about. First, the data used in this study come from industry documents. While the CIRs do provide information about the methods used for making measurements of different cigarette parameters, we had no way of verifying the accuracy of the measurements reported for any brand with the exception of reported FTC machine measured tar and nicotine levels, which do appear to match those found in FTC reports. Second, our analytic strategy sampled CIRs available in different years and then within the sampled reports we chose to focus on a limited number of cigarette brands. In this study, we have narrowly focused on comparisons of six popular filtered and three popular non-filtered brands from the 1960s. It is possible the results reported here could change had we included a larger sample of CIRs and wider selection cigarette brands and brand styles. Third, while this study does not take into consideration how different design features of cigarettes or changes in design parameters may have influenced actual smoking behaviors of consumers, the simple fact that there is less tobacco in a filtered cigarette calls into question the presumption that putting a filter tip on the cigarette was solely responsible for the lowered health risks of smoking^14-24^.

### Implications

One might wonder why we found it worthwhile to revisit the question of whether having a filter on a cigarette provides any health advantage, since virtually all people currently smoking today smoke a cigarette with a filter tip^5^. We believe there are several justifications for such inquiry. First, if there is no obvious health advantage of a cigarette filter itself one has to question why manufacturers continue to use them, since adding a filter tip incurs a higher cost in manufacture^15^. One plausible explanation for continuing to use a filter on a cigarette is that the public, even today, believes that a filter on a cigarette offers a slight health advantage compared to a cigarette without a filter^9-13^. Perpetuating this myth is not only unfair to consumers, but it diverts attention from informing the public about other cigarette design features that might actually influence disease risk such as how much tobacco is burned. As a case in point, a national survey found that people who smoke American Spirit cigarettes, a brand marketed by R.J. Reynolds Tobacco Company with terms like ‘tobacco and water’ and ‘natural’, and imagery of a Native American with a ceremonial tobacco pipe, were much more likely to rate their cigarettes as less harmful compared to other cigarette brands, even though American Spirit cigarettes contain more tobacco by weight compared to most other cigarettes^36^. It is plausible that, for some, the presence of the filter and misperceptions about lower health risks of filtered cigarettes have contributed to smoking initiation and to continued smoking among those who would have otherwise quit.

A second concern is that cigarette filters may expose people to different risks not found with a non-filtered cigarette. For example, cellulose acetate filters carry a unique health burden through the release of inhalable fibers into the airways^1,37^. The filter also changes the particle size distribution of cigarette smoke and therefore may also contribute to people inhaling more deeply and exposing them to smaller tar particles which can penetrate deeper into the airways^24,37-41^. Also, cigarette filters do not easily biodegrade, which makes them a common source of environmental pollution, particularly hazardous to aquatic life^42^.

## CONCLUSIONS

This study shows that various design features of popular filtered and non-filtered brands changed between 1960 and 1990. The observed reduction in tobacco weight among filtered brands was perhaps the most salient design change observed in terms of health risks, since the presumed lower health risk of filtered cigarettes may be due to less tobacco burned and not the filter itself. Given the potential public misperceptions about the purported effect of filters on lowering health risks of smoking, we suggest that product regulators request manufacturers to produce evidence to support the continued use of filters, assuming that all other design features are kept the same. Furthermore, cigarettes come in a wide variety of sizes, from long and slim cigarettes to those that are short and wide^5,43^. Standardizing the design of cigarettes in terms of cigarette stick length and circumference, tobacco weight, blend characteristics (and perhaps other features), might help to address consumer misperceptions about the relative health implications of different brands and brand styles. Future studies are recommended to evaluate the impact of product standardization and even the banning of filters, on consumer product perceptions, smoking behaviors, and toxicant exposure.

## Data Availability

The data supporting this research can be found in the Supplementary file.
